# Reconfigurable Assembly of Planar Colloidal Molecules via Chemical Reaction and Electric Polarization

**DOI:** 10.34133/research.0490

**Published:** 2024-01-30

**Authors:** Xi Chen, Xianghong Liu, Mohd Yasir Khan, Zuyao Yan, Dezhou Cao, Shifang Duan, Lingshan Fu, Wei Wang

**Affiliations:** ^1^ College of Materials and Chemistry & Chemical Engineering, Chengdu University of Technology, Chengdu, Sichuan 610059, China.; ^2^ School of Materials Science and Engineering, Harbin Institute of Technology (Shenzhen), Shenzhen, Guangdong 518055, China.

## Abstract

Colloidal molecules, ordered structures assembled from micro- and nanoparticles, serve as a valuable model for understanding the behavior of real molecules and for constructing materials with tunable properties. In this work, we introduce a universal strategy for assembling colloidal molecules consisting of a central active particle surrounded by several passive particles as ligands. During the assembly process, active particles attract the surrounding passive particles through phoresis and osmosis resulting from the chemical reactions on the surface of the active particles, while passive particles repel each other due to the electric polarization induced by an alternating current (AC) electric field. By carefully selecting particles of varying structures and sizes, we have assembled colloidal molecules of symmetric and asymmetric dimers, trimers, and multimers. Furthermore, the coordination number of these colloidal molecules can be regulated in real time and in situ by tuning the interaction forces between the constituent particles. Brownian dynamics simulations reproduced the formation of the colloidal molecules and validated that the self-assembly arises from chemically induced attraction and electrical dipolar repulsion. This strategy for reconfigurable colloidal assemblies poses the potential for designing adaptive micro-nanomachines.

## Introduction

Self-assembly is the process of assembling building blocks into ordered structures with exquisite complexity and functionality [1–3]. Recently, assembled colloidal clusters, commonly referred to as “colloidal molecules”, have garnered much attention due to their advantages for both fundamental research and practical applications [4,5]. Due to their appropriate size to microscopic observations and their easy accessibility in experiments, colloidal molecules offer a valuable model system for testing and validating theoretical models in crystal nucleation and glass transitions [6–8]. Moreover, because their sizes are comparable to the wavelengths of light, colloidal molecules can interact with light, resulting in unique plasmonic and photonic properties. Consequently, colloids have been widely utilized as building blocks for photonics [9,10], electronics [11,12], sensors [13,14], and microlens [15]. Furthermore, some colloidal molecules move autonomously, paving the way for the development of intelligent micro- and nanorobotic systems [16–19].

Colloidal assembly can be divided into 2 main categories: equilibrium self-assembly and out-of-equilibrium self-assembly [20]. In equilibrium self-assembly, colloidal system reaches a thermodynamically stable state dictated by minimizing the free energy, either locally or globally. Many methods have used this principle to fabricate colloidal molecules, including depletion interaction [21,22], capillary condensation [23], electrostatic interactions [24,25], colloidal fusion [26,27], coalescence [28,29], emulsion polymerization [30,31], and geometric confinement [32,33]. However, most of the assembled structures obtained through these methods are static and cannot be easily disassembled and reassembled [34–36]. In contrast, because out-of-equilibrium assembly occurs under energy dissipation, the assembled structures within this system can escape the lowest thermodynamic traps, thus preventing the system from reaching equilibrium. This type of assembly relies on the energy input from the environment, thereby exhibiting dynamic and adaptable characteristics under external stimuli, a phenomenon also known as active colloidal self-assembly.

The basic building blocks of active colloidal self-assembly are typically active colloids, which are colloidal particles capable of converting external energy, such as chemical [37,38], electrical [39], acoustic [40], optical [41], and magnetic [42] energy, into autonomous motion. In particular, the use of electric fields has shown promise in forming active colloidal self-assembly with reversibility and reconfigurability through electric polarization [43–45]. However, the formation of colloidal molecules under electric fields often requires specific combinations of colloid sizes [19,44], known as size selectivity, which impede the preparation of colloidal molecules with a large ranges of sizes. Alternatively, chemically active particles capable of converting chemical energy into motion via chemical reactions can be employed as building blocks, typically circumventing the size selection requirements for colloidal assembly [46–48]. However, it remains a challenge to precisely regulate the coordination number of the colloidal molecules in real time and to reconfigurably assemble colloidal molecules made of colloids of a wide range of sizes.

**Fig. 1.**
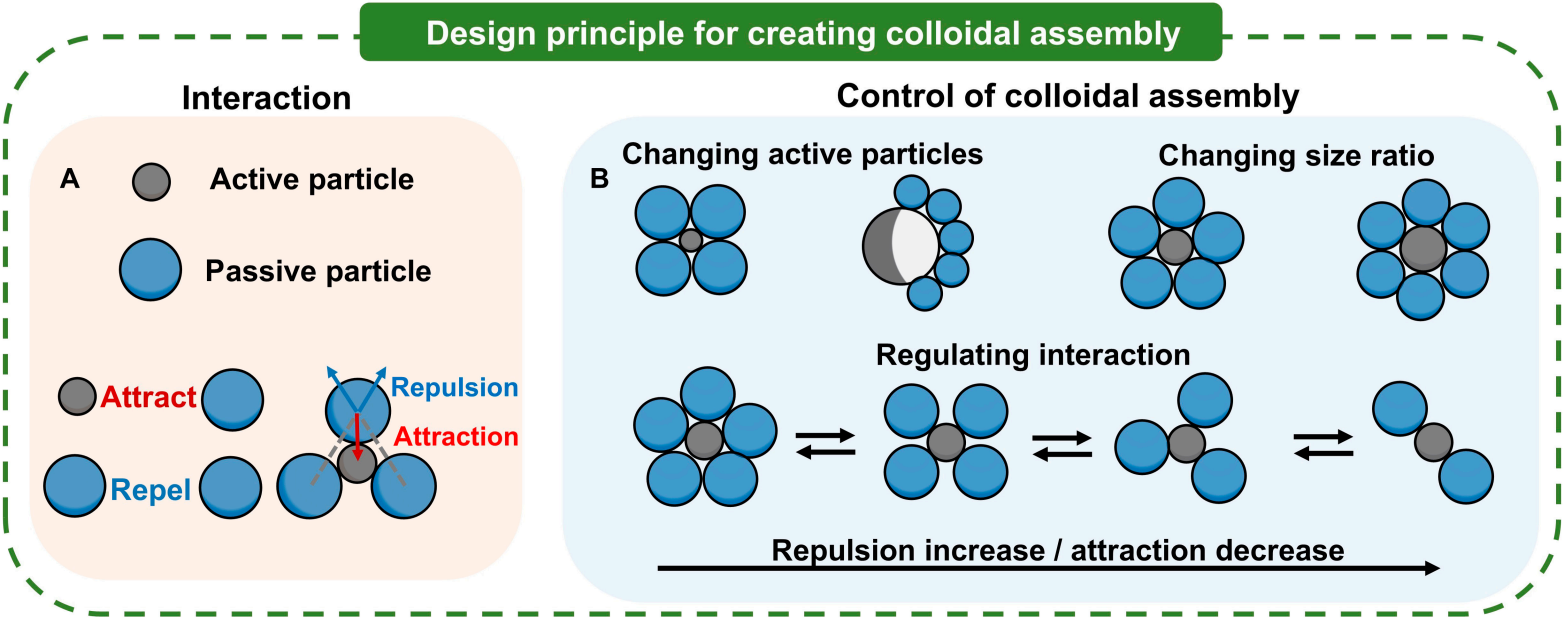
Design principles of our strategy for creating colloidal assembly. (A) Formation of colloidal assembly by attractive and repulsive interaction between active and passive particles. (B) Control of colloidal assembly by regulating experimental parameters.

In this work, we utilized a hybrid approach combining chemical and electric fields for the reversible assembly of colloidal molecules and for their in situ regulation. The resulting colloidal molecule consists of a central microsphere surrounded by a few ligand microspheres (Fig. 1A). The central particles are chemically active, which attract the ligand particles via phoresis and osmosis caused by concentration gradient. The ligand particles repel each other through the repulsive dipolar interaction caused by electric polarization under alternating current (AC) electric field. By tuning attractive and repulsive interactions, the diverse assembly with high reconfigurability can be obtained in Fig. 1B. To be more specific, an isotropic or Janus central active particle leads to colloidal molecules of symmetric and asymmetric configurations, respectively. Additionally, modifying the size ratio between the central and ligand particles allows for the formation of colloidal molecules with varying coordination numbers. Furthermore, the coordination numbers can be fine-tuned in real time by regulating the repulsive or attractive interactions among particles. This work demonstrates a strategy for colloidal assembly, which offers possibility of fabricating materials with adaptive and reconfigurable structures.

## Results

### Formation of colloidal assembly

**Fig. 2.**
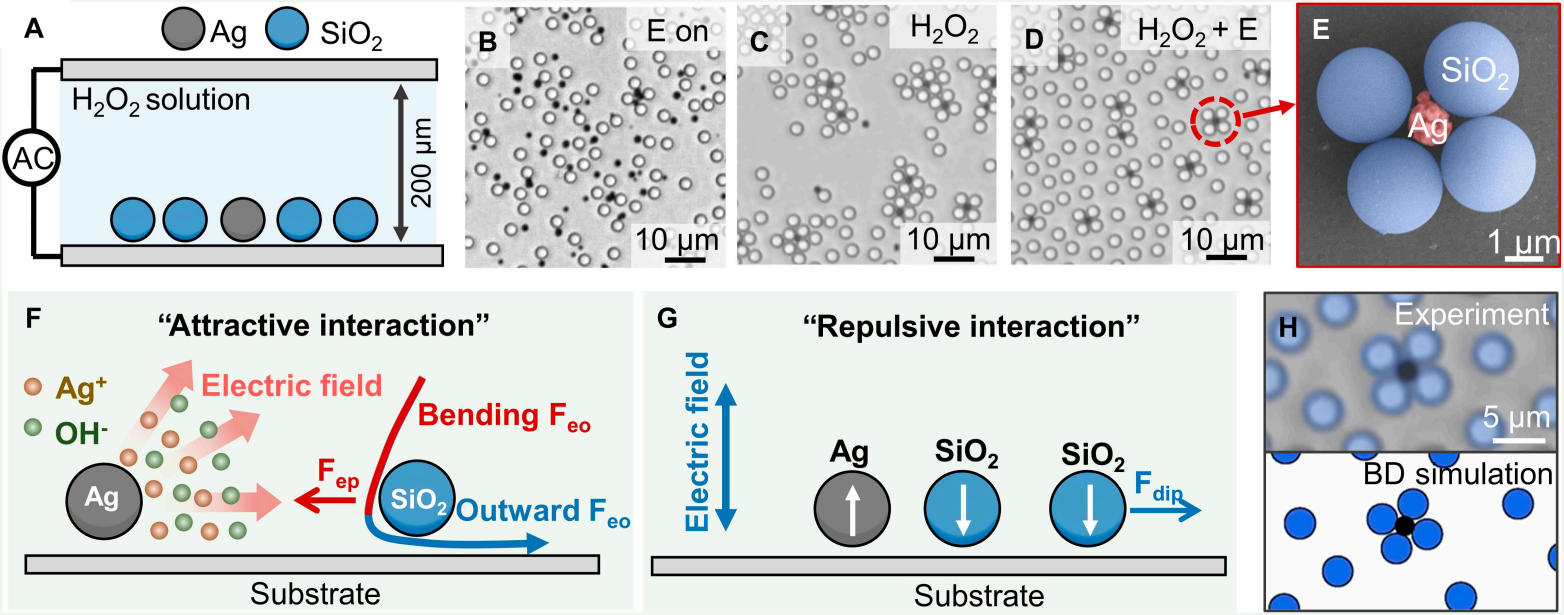
Assembly of colloidal molecules. (A) Experimental setup for the assembly between Ag and SiO_2_ particles. (B) Behaviors of Ag and SiO_2_ particle under AC electric field of 1 MHz and 10 V_pp_. (C) SiO_2_ particle assembling on the Ag particle in the solution of 0.01 wt% H_2_O_2_. (D) Formation of AB_4_ colloidal molecule under AC electric field and H_2_O_2_ solution. (E) Scanning electron microscope (SEM) of the formed AB_4_ colloidal molecule. (F) Schematic showing that an Ag particle releases Ag^+^ and OH^−^ in H_2_O_2_, and interacts with a passive SiO_2_ particle via phoresis (F_ep_) and osmosis (F_eo_). (G) Schematic showing that an active Ag particle interacts with a passive SiO_2_ particle via electrical dipolar attraction, and 2 SiO_2_ particles repel each other via electrical dipoles, in an electric field. The white arrows represent electrical dipoles. (H) Assembled AB_4_ colloidal molecule under an optical microscope (top, with fake colors) and in a Brownian dynamics (BD) simulation (bottom).

To demonstrate our strategy to prepare colloidal molecules, we first used active silver particle with a diameter of 1.5 μm as the central particle, and SiO_2_ particles with a diameter of 3 μm as the ligand particles. The detailed characterizations of these particles are shown in Figs. S1 to S3. Our experimental setup (Fig. 2A) involved sandwiching the colloidal suspension between an indium tin oxide (ITO)-coated glass substrate with a sealed experimental chamber of 200 μm in height. When an AC electric field of 1 MHz and 10 V_pp_ was applied to the colloidal solution system, there is no formation of colloidal assembly between Ag particles and SiO_2_ particles (Fig. 2B). When hydrogen peroxide was added to the experimental system without applying an electric field, the Ag particles showed a stronger attraction toward SiO_2_. The clusters grew in size over time until all neighboring SiO_2_ particles were collected by Ag, creating clusters in which Ag particles were surrounded by multiple layers of SiO_2_ (Fig. 2C). Upon applying an AC electric field of 1 MHz and 10 V_pp_, SiO_2_ particles began to repel each other, forming a planar close-packed core–shell cluster where one Ag colloid was surrounded by 4 SiO_2_ microspheres (Fig. 2D). It is a colloidal molecule of AB_4_ configuration, where A represents the central particles and B represents the ligand particles. By applying an additional 8-V direct current (DC) electric field, such colloidal molecules move toward the substrate due to electrophoresis and then are fixated on the substrate caused by van der Waals force [49]. Some assembled colloidal molecules (Fig. 2E) remained intact after removing the top ITO-coated glass and cleaning it with deionized water.

The attractive interaction for this colloidal assembly between active Ag particles and passive SiO_2_ particles probably comes from the phoretic and osmotic effect induced by chemical reaction on the surface of the Ag particle in the H_2_O_2_ solution. As shown in Fig. 2F, Ag was oxidized by H_2_O_2_ to produce Ag^+^ and OH^−^. A chemical gradient is then formed around the Ag particle. OH^−^ diffuse faster than do Ag^+^ (the diffusion coefficient of OH^−^ and Ag^+^ is 5.27 × 10^−9^ and 1.65 × 10^−9^ m^2^·s^−1^, respectively [50]). An electric field spontaneously forms to maintain the charge neutrality in the bulk solution. This self-generated electric field can induce outward electroosmotic force (F_eo_) in the electric double layer of the negatively charged substrate and inward electrophoresis (F_ep_) for the negatively charged SiO_2_ passive particles [51]. Close to the active particles, the electroosmotic flow bends downward to maintain fluid continuity, resulting in additional attraction for the passive particles [51,52]. Therefore, the osmotic and phoretic effects assemble passive particles onto the active particle (see Fig. 2F for an illustration of the various forces). The repulsive interaction during the colloidal assembly between passive SiO_2_ particles comes from the electric polarization of SiO_2_. Figure 2G presents the schematic of the dipolar interactions between SiO_2_ passive particles in an AC electric field. SiO_2_ particles were polarized in the same direction under an AC electric field, leading to dipolar repulsion between SiO_2_ particles. Note that the Ag and SiO_2_ particles are polarized in the opposite directions under our experimental conditions (Fig. S4), potentially leading to dipolar attraction. However, we found that such a dipolar attraction force between Ag and SiO_2_ is too weak to be the primary mechanism for assembling colloidal molecules (Fig. 2B and Movie S2).

To validate that the observed colloidal assembly is caused by the above attractive and repulsive interactions, we performed Brownian dynamics (BD) simulations based on over-damped Langevin equations (see details in Materials and Methods). The central and ligand particles were represented as black and blue particles in the BD simulation, respectively. All the particles were subject to thermal fluctuations and pairwise interactions that take into consideration phoresis, osmosis, and dipolar interactions. In this simulation, the attractive force caused by phoresis and osmosis can be described by the following phenomenological model [51]:

$$F_{\mathrm{att}}=-\frac{1}{\lambda }U_{0}^{\prime }e^{-r/\lambda }+\alpha_{a}\mu_{p}U_{0}^{D}\frac{a^{2}}{r^{2}}\;$$ (1)

where *r* is the center-to-center distance between active and passive particles, $U_{0}^{\prime }\;$ is a coefficient related to the bending of osmotic flows at short distances, *λ* is a parameter of how fast the interaction decays with distance, *α* is related to the ionic flux of active particle, *μ_p_* is the mobility of passive particle, *a* is the radius of active particle, and $U_{0}^{D}$ is a coefficient related to the magnitude of the phoretic/osmotic force that is proportional to self-generated electric field. The magnitude of this electric field follows [53]:

$$E=\frac{k_{B}T}{Ze}\cdot \frac{D_{+}-D_{-}}{D_{+}+D_{-}}\cdot \frac{\nabla c}{c}$$ (2)

where *k_B_* is the Boltzmann constant, *T* is the temperature, *Z* is the number of charges, *e* is the elementary charge, *c* is the local ionic concentration, and *D* is the diffusion coefficient of ions.

The repulsive force caused by dipolar interaction between SiO_2_ particles is governed by [49,54]:

$$F_{\mathrm{dip}}=\frac{3}{4}\pi \varepsilon_{m}R^{2}{\left|K\right|}^{2}{E_{\mathrm{rms}}}^{2}{\left(\frac{2R}{r}\right)}^{4}$$ (3)

where *r* is the center-to-center distance, $E_{\mathrm{rms}}=\frac{V_{\mathrm{pp}}}{2\sqrt{2}H}$ is the root mean square of the applied field, *V_pp_* is the peak-to-peak voltage (in volts), *H* is the height of the experimental chamber, and *K* is the polarization coefficient, also known as the Clausius–Mossotti factor.

Figure 2H and Movie S1 present the colloidal molecule reproduced by BD simulations. This result confirms that the phoretic/osmotic force, caused by the chemical reaction on the surface of active Ag particles, and the dipolar force, caused by the electric polarization of passive SiO_2_ particles, result in the planar colloidal assembly.

### Control of colloidal assembly

#### Regulating the configurations and shapes of colloidal molecules

**Fig. 3.**
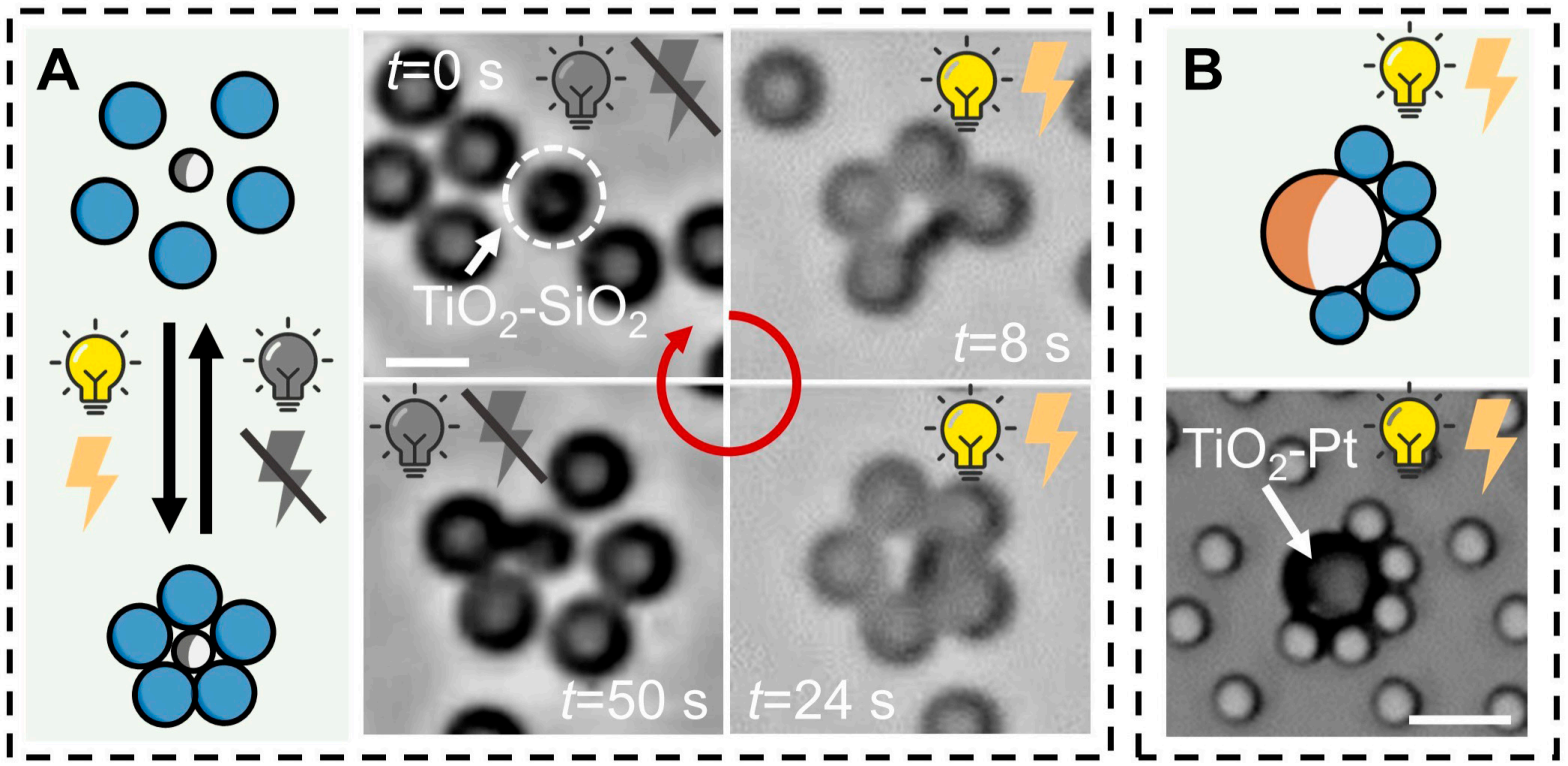
Colloidal molecules of symmetric (A) or asymmetric (B) configurations. (A) Colloidal molecules with symmetric configuration formed by five 3-μm SiO_2_ microspheres attracting to a 2-μm active TiO_2_–SiO_2_ particle. At *t* = 1 s, UV light and AC electric field were turned on. At *t* = 44 s, UV light and AC electric field were turned off. (B) Colloidal molecule with asymmetric configuration formed by several 3-μm passive SiO_2_ particles attracted to a 10-μm active TiO_2_–Pt Janus microsphere. Experimental condition: 1.5 wt% H_2_O_2_, 563 mW/cm^2^ UV light, and AC electric fields of 10 V_pp_ and 100 kHz. Scale bars, 5 μm.

Our strategy, which relies on chemical reaction and electric polarization, can be also applied to active particles other than Ag. To further endow attractive interaction of active and passive particle with switch on/off functions, we utilized light-responsive 2-μm SiO_2_ microspheres half coated with TiO_2_ (i.e., TiO_2_–SiO_2_) and 3-μm SiO_2_ particles as the central and ligand particles, respectively. The reason for choosing Janus TiO_2_–SiO_2_ as the photoactive particles to demonstrate our control capacity is its simple preparation process, which only requires sputtering a layer of TiO_2_ on one side of SiO_2_. The uniformity and monodispersity of Janus TiO_2_–SiO_2_ produced through the above physical method are better than those of chemically synthesized isotropic core–shell SiO_2_@TiO_2_. The colloidal molecule with full coordination number can be also formed by choosing Janus TiO_2_–SiO_2_ as active particles [47]. This is likely due to the relative long-range chemical gradient induced by the photocatalytic reaction on the surface of TiO_2_. Consequently, the chemical gradient can also be generated on the passive SiO_2_ side, allowing particles around this side to be also attracted. Due to the ultraviolet (UV) activation of TiO_2_–SiO_2_ particles, the attractive force can be switched on by turning on UV light. One typical formation process is shown in Movie S3, where 1.5 wt% H_2_O_2,_ 563 mW/cm^2^ UV light, and 10 V_pp_ and 100 kHz AC electric field were used. The whole field of view showing multiple colloidal molecules of similar structures is given in Fig. S5. Upon exposure to UV light, electron–hole pairs are generated at the TiO_2_ side, which facilitates the electrochemical catalytic decomposition of H_2_O_2_, leading to a chemical gradient. The resulting gradient causes SiO_2_ particles to move toward TiO_2_–SiO_2_, and more detailed mechanism of assembly will be discussed in Discussion. As depicted in Fig. 3A, initially, all particles exhibited Brownian motion. After we turned on the UV light and AC electric field, a colloidal molecule with an AB_5_ structure gradually emerged, where one TiO_2_–SiO_2_ active particle was surrounded by a compact layer of SiO_2_ tracers. When light and electric fields were turned off, the assembled structure dissolved, and all particles returned to Brownian motion.

Colloidal molecules with asymmetric configurations can also be obtained by using a Janus active particle that interacts with nearby tracers significantly differently between its 2 hemispheres. TiO_2_–Pt Janus microspheres made by half coating TiO_2_ microspheres with a thin Pt layer is taken as an example in Fig. 3B. When exposed to UV, holes and electrons are generated in TiO_2_ [55,56]. The photogenerated holes facilitate the oxidation of H_2_O and O_2_, producing H^+^ during the process, while the photogenerated electrons move to the Pt sides reducing H^+^ to H_2_. A local electric field was created and pointed from the TiO_2_ side to the Pt side. Therefore, negatively charged SiO_2_ tracers were electrostatically attracted to accumulate to the TiO_2_ side and repelled from the Pt side [17]. In addition, because of the electrical dipolar repulsion, the SiO_2_ tracers were evenly distributed along the edge of the TiO_2_ hemisphere and evenly separated from each other. Note that an isotropic, closed packed SiO_2_ layer was formed around the TiO_2_–SiO_2_ microsphere in Fig. 3A, while an asymmetric layer was formed for TiO_2_–Pt in Fig. 3B. This difference is possibly because that SiO_2_ hemisphere of TiO_2_–SiO_2_ was not a photocathode like the Pt cap in a TiO_2_–Pt that repelled SiO_2_ tracers.

**Fig. 4.**
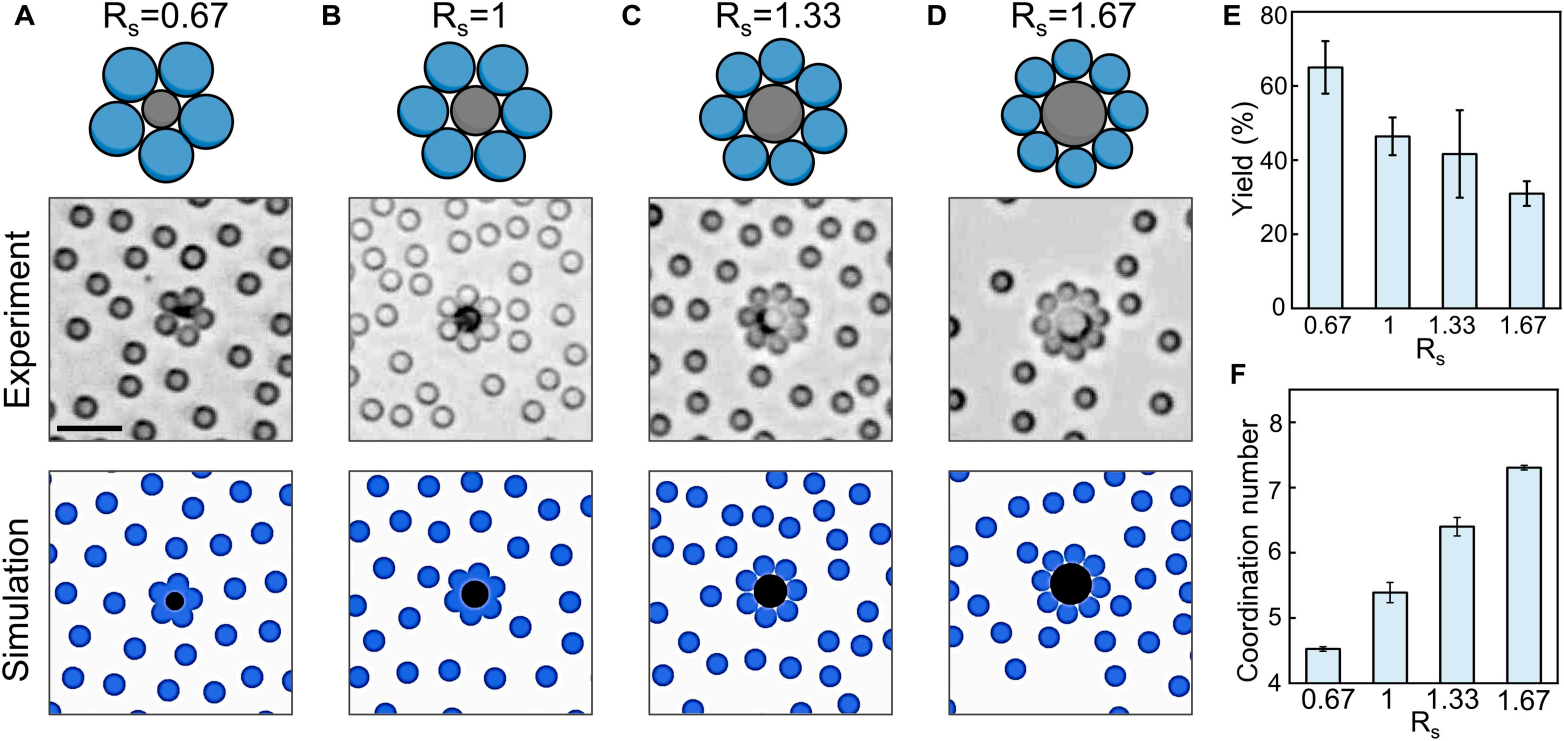
Colloidal molecules with different coordination numbers. (A) Pentagonal AB_5_ at R_s_ = 0.67. (B) Hexagonal AB_6_ at R_s_ = 1. (C) Heptagonal AB_7_ at R_s_ = 1.33. (D) Octagonal AB_8_ at R_s_ = 1.67. Top: Schematic. Center: Optical micrographs of the colloidal molecules consisting of one TiO_2_–SiO_2_ Janus microsphere at the center and several SiO_2_ microspheres as ligands. Bottom: BD simulations. R_s_ is defined as the ratio of diameters between the active and passive particles. (E) Yield of colloidal molecule with full coordination number at different R_s_. The yield is defined as the proportion of assemblies with full coordination number relative to all assemblies. (F) Mean coordination number of colloidal molecules at different R_s_. Experimental conditions: 2-, 3-, 4-, and 5-μm TiO_2_–SiO_2_ as the central particles in (A) to (D), respectively, and 3-μm SiO_2_ microspheres as the ligand particles, under 10 V_PP_ and 100 kHz electric field and 563 mW/cm^2^ UV in 1.5 wt% H_2_O_2_ solution. Scale bars, 10 μm.

The shape of the colloidal molecules can be regulated by changing the size ratio between the active TiO_2_–SiO_2_ particles and passive SiO_2_ particles. We define the ratio of the diameters between the active and passive particles as R_s_. Figure 4 and Movie S4 demonstrate the formation of colloidal molecule with pentagonal (AB_5_), hexagonal (AB_6_), heptagon (AB_7_), and octagonal (AB_8_) shapes at R_s_ values of 0.67, 1, 1.3, and 1.67, respectively. These experimental results were faithfully reproduced in BD simulations by tuning the size ratio of active and passive particles. We also have a statistical analysis of colloidal molecule assembly in Fig. 4E and F. Figure 4E presents the yield of colloidal molecules with full coordination number at different R_s_. The yield is defined as the proportion of colloidal molecules that have full coordination number relative to all assemblies. As shown in Fig. 4E, the yield remains above 30% for all R_s_. However, it decreases as R_s_ increases, because larger active particles require more passive particles to achieve full coordination, resulting in a lower yield of fully coordinated assemblies. The distributions of colloidal molecules with different coordination numbers at different R_s_ are shown in Fig. S6. The reason that not all of colloidal molecules have full coordination number is due to the limited range of attraction caused by the chemical gradient. When there are not enough passive particles in close to the active particles, colloidal molecules with full coordination number cannot form. Therefore, the coordination number of the assemblies can also be regulated by changing the ratio of active to passive particles. As shown in Fig. S7, as the density of passive particles decreases, the coordination number of the assemblies decreases. Additionally, we analyzed the mean coordination number of assemblies at different R_s_. While the yield of fully coordinated assemblies decreases with larger active particles, the mean coordination number increases (Fig. 4F).

#### Regulating the coordination numbers of colloidal molecules in real time

**Fig. 5.**
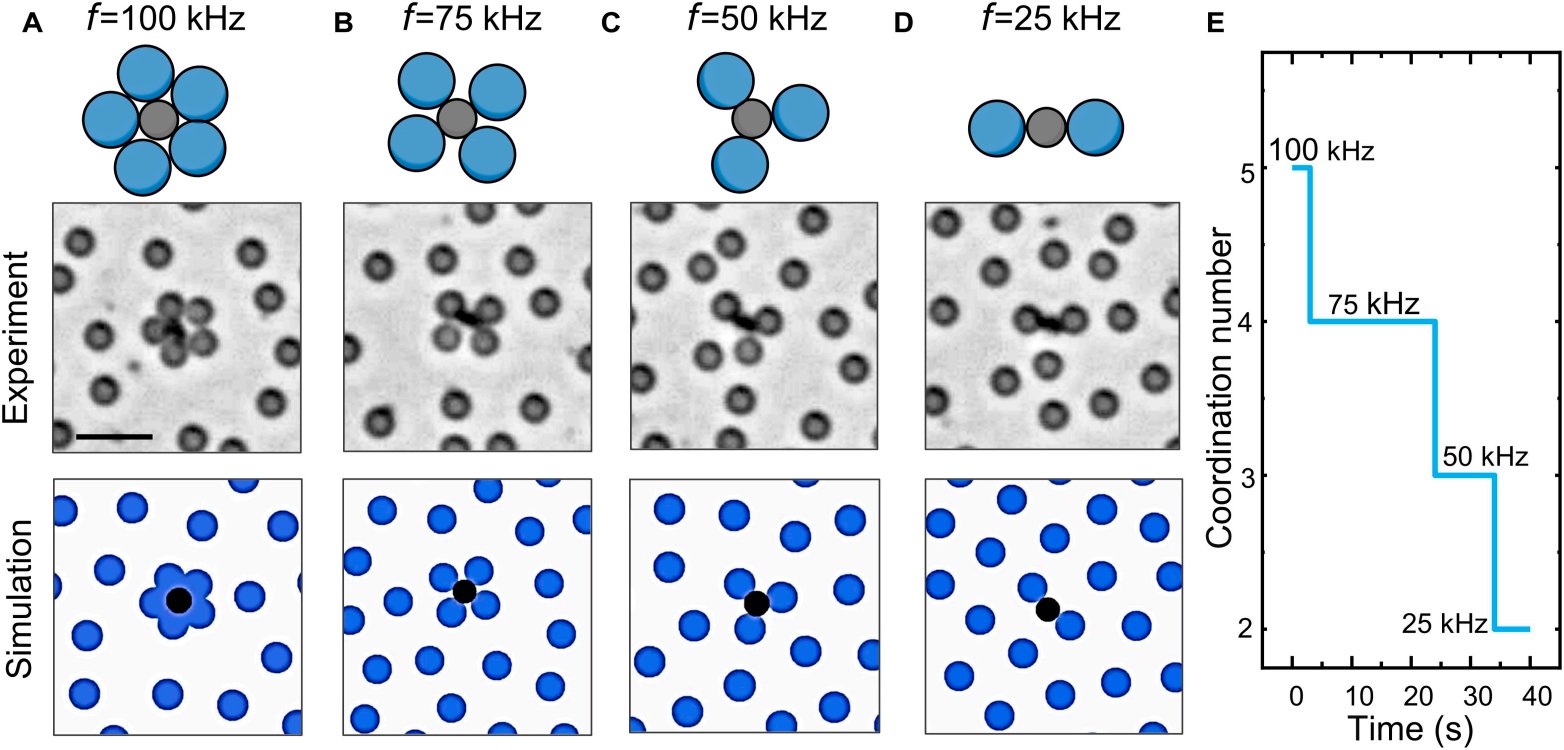
Regulating the coordination number of colloidal molecules in real time by tuning the frequency of the AC electric fields, *f*. (A) AB_5_ colloidal molecule at 100-kHz AC electric field. (B) AB_4_ colloidal molecule at 75-kHz AC electric field. (C) AB_3_ colloidal molecule at 50-kHz AC electric field. (D) AB_2_ colloidal molecule at 25-kHz AC electric field. Top: Schematics. Center: Optical micrographs. Bottom: BD simulations. (E) Coordination number of the colloidal molecules in (A) varied in real time by gradually decreasing the driving frequency of the AC electric fields over time. Experimental conditions: 2-μm TiO_2_–SiO_2_ and 3-μm SiO_2_ under 10 V_PP_ electric field and 563 mW/cm^2^ UV in the 1.5 wt% H_2_O_2_ solution. Scale bars, 10 μm.

Because our strategy is based on 2 independent mechanisms, the magnitude of the attractive and repulsive forces can be independently adjusted in situ by regulating the parameters of the chemical field and electric field, respectively. For example, the repulsive force between the SiO_2_ particles can be regulated by changing the frequency of the electric field. As described in Eq. 3, the repulsive force caused by the induced electrical dipoles is related to K, which varies over the driving frequency [see Fig. S4 for a plot of Re(K) as a function of frequency]. Figure 5 shows that, as the frequency of the electric field decreases from 100 kHz to 25 kHz, Re(K) of SiO_2_ increases from 0.8 to 1.0, increasing the dipolar repulsion among SiO_2_ particles, and thus decreasing the coordination number of the formed colloidal molecules from AB_5_ to AB_2_. The results can be reproduced in BD simulations by adjusting the magnitude of the repulsive force caused by dipolar interaction. It should be noted that although modulating the frequency of electric field can regulate the coordination number of colloidal molecules in situ, there is a slight deviation in the characteristic frequency during the coordination number transition process for different colloidal molecules (Fig. S8). This may be due to slight differences in the surface morphology and catalytic capabilities of the prepared Janus TiO_2_–SiO_2_ particles, which leads to slight differences of the chemically induced attraction and electrically induced repulsion interactions. The magnitude of the attractive force can also be regulated by changing the intensity of the UV light: As the light intensity decreases, the attractive force weakens, resulting in a decrease in the coordination number (Fig. S9).

## Discussion

Taking advantage of independently tuning the attractive and repulsive interaction among particles, our strategy addresses the limitations associated with existing methods for assembling colloidal molecules. A comparison is listed in Table. Unlike colloidal self-assembly in the equilibrium system (orange shading in Table), our strategy allows for the reversible assembly of colloidal molecules. Compared to colloidal assembly in the nonequilibrium system like using chemical field or electric field (blue shading in Table) to assemble colloidal molecules, our strategy (green shading in Table) combines both fields to avoid size selectivity, providing more choices for the size of particles to achieve self-assembly. Moreover, our strategy enables the formation of non-close-packed colloidal molecules structures, similar to the real molecules.

**Table.**
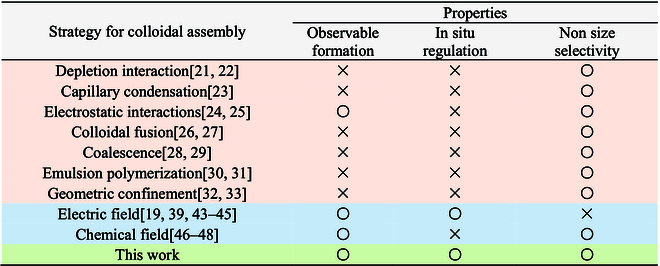
A comparisons of the various methods for assembling colloidal molecules. “○” represents that the method satisfies a particular property, while “×” does not. Orange and blue shadings correspond to equilibrium and nonequilibrium assembly, respectively.

To provide a clean explanation of the assembling mechanism in this paper, we further discuss the repulsive and attractive interactions between active and passive colloids in this section. The repulsive interactions between passive particles during assembly are well understood. The same direction polarization of particles induced by electric fields leads to repulsive forces, which is a well-established theory [57]. Regarding the source of the attractive forces caused by the electric field in the assembly, the polarization directions of the active and dielectric particles differ, which could lead to attraction. However, our experiment shows that this attraction is not strong enough to form the colloidal molecule (Fig. S10A). Under only a chemical field without an electric field, the attraction force becomes significant (Fig. S10B).

Therefore, the attractive forces primarily come from chemical reactions on the surface of active colloids. However, the nature of the force exerted by the catalytic decomposition of H_2_O_2_ on the surface of TiO_2_ particles, which influences the surrounding passive particles, is still debated—whether it arises from neutral chemical gradients or ionic chemical gradients remains uncertain [58]. One proposal suggests that the attractive interaction between photocatalytic particles and passive particles is due to diffusiophoresis caused by neutral chemical gradients [47]. Under light exposure, photocatalytic particles consume H_2_O_2_ in the solution to generate O_2_, creating local gradients of H_2_O_2_ or O_2_, which drive fluid motion. However, neutral self-diffusiophoresis still needs definitive proof, as ion intermediates might also be produced during the decomposition of H_2_O_2_, leading to electrokinetic effects, such as electrophoresis and electroosmosis. For instance, it has been reported that photocatalytic surfaces such as BiVO_4_ can decompose H_2_O_2_, resulting in ion diffusion on the formation of a self-generated electric field [59].

It is worth noting that colloidal molecule can also self-propel. Figure S11 shows that colloidal molecule with a coordination number of 3 can self-propel, as its structure was made asymmetric by random thermal noises. The self-propulsion was often observed at low coordination numbers of assemblies, because the drag force acting on the colloidal assembly is weaker than those assemblies with large coordination number. The results demonstrate that active colloids can not only induce assembly but also enable the colloidal assembly to move, which is helpful for the design of micro/nano-machine systems.

We mainly observed the formation of planar colloidal molecules in this work, because the particles settled down to the bottom surface of the experimental chamber due to the density mismatch between the particles and the aqueous solution. However, real molecules are not always planar and often have 3-dimensional (3D) configurations, making it necessary to investigate 3D colloidal assembly. In this active colloidal system, when the population density of the ligand particles was increased or ligand particles of different sizes were used, 3D assembly structures were formed due to the out-of-plane attractive interactions among electrically polarized colloids [43]. An example of the 3D colloidal molecules formed this way is given in Fig. S12. More diverse 3D assembly structures can be achieved by designing assembly units with specific structures. For example, assembly units with special structures, such as dimers, can form chiral assemblies caused by dipolar interaction under an electric field [45]. For assemblies under chemical stimuli, Janus hematite cubes at vertical orientation when exposed to light can form 3D micro-rotors by assembling surrounding cubes due to the chemically induced hydrodynamic flow [60]. Cube-shaped central particles can also form 3D colloidal assemblies through the action of DNA linkers [61]. However, the abovementioned 3D assembly structures still face limitations in dynamic in situ control of the assembly units. In principle, the combination of chemical and electric fields proposed in this paper could achieve in situ control of 3D assemblies. However, we are currently limited by the ability to fabricate assembly units that are both chemically active and have special morphologies and structures. Future efforts will explore methods such as chemical synthesis or photolithography to prepare these assembly units, enabling us to control the attractive and repulsive forces between them using chemical and electric fields, thereby giving us the ability to design more diverse 3D assembly structures. This aspect will be further studied in our future work.

In summary, we present a reconfigurable strategy for constructing colloidal molecules via chemical reactions and electrical polarization. The assembled colloidal molecules consist of a chemically active colloidal particle at the center and a few surrounding ligand particles in a core–shell configuration. The attraction between the central and the ligand particle comes from phoresis and osmosis that originates from the chemical reactions occurring on the surface of the central particle, while the interparticle repulsion induced by the electric dipoles of a ligand particle governs the final assembly. Colloidal molecules of symmetric or asymmetric structures can be obtained by choosing isotropic or Janus central particles, respectively. The shape (coordination number) of the colloidal molecules can be controlled by tuning the size ratios of the central and ligand particles. Moreover, the real-time regulation of the coordination number of colloidal molecules is achieved by tuning the driving frequency of AC electric fields and light intensity. This strategy for preparing colloidal molecules could aid the design and fabrication of active materials with tunable structures.

## Materials and Methods

### Details of the microspheres used in experiments

#### Details of SiO_2_ particles

SiO_2_ particles of 2, 3, and 5 μm diameter were bought from Huge Biotechnology, China. The obtained SiO_2_ particles are highly monodispersed.

#### Preparation of Ag microspheres

Ag microspheres were prepared by mixing 0.15 M silver nitrate solution [analytical reagent (AR), Titan, China] and 0.09 M ascorbic acid solution (AR, Aladdin, China) with 0.5 wt % polyvinyl pyrrolidone (AR, Aladdin, China) to react at 0 °C under continuous stirring. The prepared Ag particle is monodispersed, and its size is about 1.5 μm. The typical morphology of the prepared Ag particle is shown in Fig. S1.

#### Preparation of Janus SiO_2_–TiO_2_

We used the drop-casting method to prepare a monolayer of SiO_2_ (2 μm in diameter) on glass slide. Specifically, a certain amount of SiO_2_ was suspended in ethanol (E111994 99.5%, Aladdin, China) and dispersed by ultrasound. The suspension was then drop-casted onto the glass slide. The monolayer of SiO_2_ was coated with a 120-nm TiO_2_ layer via magnetron sputtering (dual-cavity magnetron sputtering equipment, KYKY-500CK-500ZE). The as-prepared SiO_2_–TiO_2_ Janus microparticles were annealed at 550 °C for 2 h in air. After being annealed, the Janus microparticles were released from the glass slides by ultrasonication and then resuspended in deionized water. The typical morphology of the prepared SiO_2_–TiO_2_ Janus particles is shown in Fig. S2.

#### Preparation of Janus TiO_2_***–***Pt

TiO_2_ particles (10 μm in diameter) were prepared by a core–shell technique [62], which coats a thin (~90 nm) TiO_2_ shell around a SiO_2_ microsphere core. A monolayer of such core–shell TiO_2_ microspheres on a glass slide was prepared by the drop-casting method described above. Then, TiO_2_–Pt Janus microspheres were prepared by sputtering a 20-nm-thick Pt layer on top of the TiO_2_ monolayer. The Janus microspheres were then removed from the substrate and resuspended in deionized water for later use. The typical morphology of the prepared TiO_2_–Pt Janus particles is shown in Fig. S3.

### Experimental setup for colloidal assembly

The schematic of experimental setup is shown in Fig. 2A. The experimental chamber for AC experiments was constructed by stacking together 2 pieces of coverslips coated with 200 ± 50 nm ITO (custom-ordered, 7 to 10 Ω/cm^2^), with the ITO sides facing each other. A silicone spacer ∼200 μm thick (custom-ordered, Gracebio) was placed in between. A wave function generator (model 33210A, Keysight) was connected to both ITO slides with copper tapes, and sinusoidal waves of 10 V (peak to peak) and 100 kHz were applied. The suspension of active and passive particles was pipetted into the homemade chamber and observed from underneath with an inverted optical microscope (Olympus IX71). UV light was applied from the top. The intermittent UV light was carried out with a light-emitting diode UV light source (Thorlab M365LP1-C1, peak wavelength at 365 nm). The assembly behavior of particles was recorded by complementary metal-oxide semiconductor camera (GS3-U3-51S5C-C, Point Grey) typically at 20 frames per second.

### Brownian dynamic (BD) simulation

A 2D model was built to simulate the formation of colloidal molecules. The central particles in experiments were set as black in simulation, while the ligand particles in experiments were blue. The dynamical evolution of the colloidal particles in this work is affected by thermal fluctuations, mutual pairwise interaction, chemical fields, and the external electric field. In our model, the hydrodynamic interactions and the reaction–diffusion of chemicals were ignored. We assume Brownian particles of diameter *σ_i_* randomly scattered in a fluid of viscosity *η* under a 2D periodic boundary. Positions of each colloid i are represented by ***r***_i_(t). Following the physics of the low Reynolds number, we model our system using BD to study the assembly of the central and ligand particles in the following equation:

$$\overset{\cdot }{r_{i}}=\frac{D_{i}}{k_{B}T}\left(F_{i}+F_{i}^{\mathrm{att}}+F_{i}^{\mathrm{dip}}\right)+\sqrt{2D_{T}}\xi$$ (4)

A total of 3 forces are present between 2 colloids. ***F****_i_* = −∇*_i_U_ij_* is the pairwise hardcore volume exclusion force between any particle i and j, derived from the Weeks–Chandler–Andersen (WCA) potential *U_ij_*. $F_{i}^{\mathrm{att}}$ is the chemical force between black particle i and blue particle j. $F_{i}^{\mathrm{dip}}$ is the dipolar force between blue particle i and blue particle j. *ξ* is a Gaussian white noise process of zero mean and correlation <*ξ_i_*(*t*)*ξ_j_*(*t*^′^) >  = *δ_ij_δ*(*t* − *t*^′^). $D_{i}=\frac{k_{B}T}{\gamma_{i}}$ is the translational diffusion coefficient, where *γ_i_* = 3*πησ_i_* is the Stokes friction term, *k_B_T* is the Boltzmann factor, *T* is the temperature, and *k_B_* is the Boltzmann constant.

The hardcore interaction between the particles *i* and *j* is modeled using the WCA potential:

UijWCA=4ε0σi+σj2rij12−σi+σj2rij6+14∀rij<21/6σi+σj/20Otherwise (5)

where *r*_*ij* is the distance between any 2 particles and *ε*_0_ = *k_B_T*.

The interaction forces caused by ionic diffusiophoresis and diffusioosmosis is modeled as [51]:

$$F_{i}^{\mathrm{att}}=\varepsilon_{0}\left(Ae^{-\frac{r_{\mathrm{ij}}}{C}}+\frac{B}{{r_{\mathrm{ij}}}^{2}}\right){\hat{r}}_{\mathrm{ji}}$$ (6)

where $Ae^{-\frac{r_{\mathrm{ij}}}{C}}$ represents the short-range interaction and $\frac{B}{r^{2}}$ represents the long-range interaction. We only observed the short-range attraction between active and passive particles in the experiment, and there is no obvious observation of long-range attraction. Additionally, we included a comparison of colloidal assembly with and without long-range interaction term in the simulation. As shown in Fig. S13, the colloidal assembly structures in these 2 situations have little difference. For simplicity, we ignored the long-range term in the simulation.

The interaction forces caused by induced dipole are modeled as [49,54]:

$$F_{i}^{\mathrm{dip}}=\varepsilon_{0}\left(\frac{D}{{r_{\mathrm{ij}}}^{4}}\right){\hat{r}}_{\mathrm{ij}}$$ (7)

We simulated *N* = 405 number of Brownian particles of diameter *σ_i_*, where 400 is the number of ligand particles of diameter *σ*_*i* ∈ [1,400]_ = 3 μm and 5 is the number of central particles of diameter *σ*_*i* > 400_ = 3*a_r_* μm, where *a_r_* is a tunable multiplicative factor that decides the central particle size.

Here, the parameters A = 5 × 10^3^, C = 0.4σ_i_, D = 1 × 10^2^, and *a_r_* = 0.6 are taken to match the experiment in Fig. 2H. The non-dimensionalization is done using the basic dimensions of length *l*_0_ = *σ*_*i* ∈ [1,400]_ = 3 μm, energy *ε*_0_ = *k_B_T*, and time *τ*_0_ = 1 s, setting the viscosity $\eta =20\eta_{0}=\frac{20\tau_{0}\varepsilon_{0}}{l_{0}^{3}}=\frac{0.003\mathrm{Ns}}{m^{2}}$. The finite time step is fixed in our study as *dt* = 0.001 *τ*_0_.

## Data Availability

All data are available in the main text or the Supplementary Materials.

## Supplementary Materials

Supplementary 1 Supplementary Text Figs. S1 to S13 Movies S1 to S5 References
