# Supplementary material for: Reconfigurable Assembly of Planar Colloidal Molecules via Chemical Reaction and Electric Polarization
Source: Research (Wash D C). 2024 Jan 30;7:0490. doi: 10.34133/research.0490 (PMC11440515; doi:10.34133/research.0490)
Supplement: Supplementary 1 — Supplementary Text Figs. S1 to S13 Movies S1 to S5 References [file research.0490.f1.zip › Supplementary Material.docx]

**Supplementary Material**

Reconfigurable Assembly of Planar Colloidal Molecules via Chemical Reaction and Electric Polarization

Xi Chen^1,2^*,†, Xianghong Liu^2,^†, Mohd Yasir Khan^2^, Zuyao Yan^2^, Dezhou Cao^2^, Shifang Duan^2^, Lingshan Fu^2^, Wei Wang^2^*

^1^College of Materials and Chemistry and Chemical Engineering, Chengdu University of Technology, Chengdu, Sichuan 610059, China

^2^School of Materials Science and Engineering, Harbin Institute of Technology (Shenzhen), Shenzhen, Guangdong 518055, China

*Corresponding author Email: [weiwangsz@hit.edu.cn](mailto:weiwangsz@hit.edu.cn), [xichen@cdut.edu.cn](mailto:xichen@cdut.edu.cn),

† Xi Chen and Xianghong Liu contributed equally to this work.

Table of Contents

[1. Experimental section 3](#_Toc176161732)

[2. Plots of the real component of the Clausius–Mossotti factor (Re(K)) 4](#_Toc176161733)

[3. The whole field of view of colloidal molecules formed 7](#_Toc176161734)

[4. Distributions of colloidal molecules with different coordination numbers 8](#_Toc176161735)

[5. Colloidal molecules with different packing fractions 9](#_Toc176161736)

[6. Transition process of four colloidal molecules 10](#_Toc176161737)

[7. Regulating the structure of colloidal molecules 11](#_Toc176161738)

[8. Assembled behaviors under only electric field and light, respectively 12](#_Toc176161739)

[9. Self-propulsion of a colloidal molecule 13](#_Toc176161740)

[10. Self-assembly structures in three dimensions 14](#_Toc176161741)

[11. Colloidal assembly with and without long-range term of chemical attraction 15](#_Toc176161742)

[12.Supporting videos 16](#_Toc176161743)

[13. Reference 17](#_Toc176161744)

# 1. Experimental section

(1) Preparation of Ag microspheres.


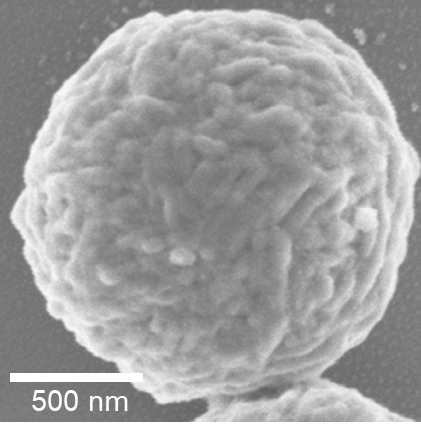


Fig. S1. Scanning electron microscope image of an Ag microsphere.

(2) Preparation of Janus SiO_2_-TiO_2_.


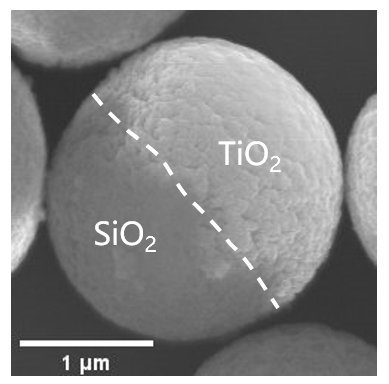


Fig. S2. Scanning electron microscope image of a SiO_2_-TiO_2_ Janus microsphere.

(3) Preparation of Janus TiO_2_-Pt.


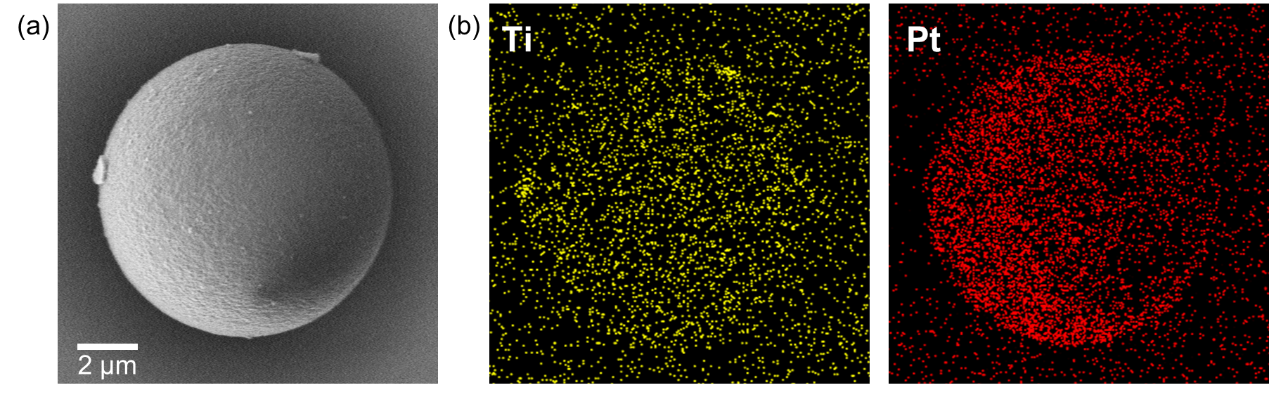


Fig. S3. a) SEM image and b) EDS of a TiO_2_-Pt Janus microsphere.

# 2. Plots of the real component of the Clausius–Mossotti factor (Re(K))

Re (K) is the real component of the Clausius–Mossotti factor, which can be solved by the following expression[1]:

|  | $K= \frac{\varepsilon_{p}^{*}-\varepsilon_{m}^{*}}{\varepsilon_{p}^{*}+{2\varepsilon}_{m}^{*}}$ | $(Eq.S1)$ |
| --- | --- | --- |
|  | $\varepsilon_{p}^{*}=\varepsilon_{p}-i\frac{\sigma_{p}}{\omega}$ | $(Eq.S2)$ |
|  | $\varepsilon_{m}^{*}=\varepsilon_{m}-i\frac{\sigma_{m}}{\omega}$ | $(Eq.S3)$ |

Where $\varepsilon_{m}$and $\varepsilon_{p}$are the dielectric permittivity of the media and the particle, respectively, and$\sigma_{m}$ and$\sigma_{p}$ are their electrical conductivity. $\omega$ is the driving frequency of the oscillating electric field.

The $\sigma_{m}$ of a 1:1 electrolyte of valence of z is:

|  | $\sigma_{m}=\sigma_{m}^{+}+\sigma_{m}^{-}=\frac{z^{2}e^{2}N_{A}c_{0}}{k_{B}T}(D^{+}+D^{-})$ | $(Eq.S4)$ |
| --- | --- | --- |

Where $+$ and $-$ correspond to the cation and anion, respectively, and $D$ is the ion diffusivity. $c_{0}$ is the ion concentration in the bulk, which can be deduced from the value of pH, and $N_{A}$ is the Avogadro number.

The $\sigma_{p}$ of a dielectric particle of radius $R$ is sum of its bulk conductivity $\sigma_{b}$ and surface conductivity $\sigma_{s}$, the latter of which is further composed of the conductivity of the Stern layer ($K_{sl}$) and that of the diffuse layer ($K_{d}$) [2–4]:

|  | $\sigma_{p}=\sigma_{b}+(K_{sl}+K_{d})/R$ | $(Eq.S5)$ |
| --- | --- | --- |
|  | $K_{d}=\frac{2\sigma_{m}}{\kappa}\left\{ \begin{matrix} \frac{D^{+}}{D^{+}+D^{-}}\left[ exp\left( -\frac{Z\tilde{\zeta}}{2} \right)-1 \right]\left( 1+3m^{+} \right) \\ +\frac{D^{-}}{D^{+}+D^{-}}\left[ exp\left( +\frac{Z\tilde{\zeta}}{2} \right)-1 \right]\left( 1+3m^{-} \right) \end{matrix} \right\}$ | $(Eq.S6)$ |
|  | $\tilde{\zeta}=\frac{e}{k_{B}T} \zeta_{p}$ | $(Eq.S7)$ |
|  | $m^{\pm}=\frac{2\varepsilon_{m}}{3\eta D^{\pm}}{(\frac{k_{B}T}{ze})}^{2}$ | $\left( Eq.S8 \right)$ |
|  | $\kappa^{-1}=\lambda_{D}=\sqrt{\frac{\varepsilon_{m}k_{B}T}{2z^{2}e^{2}n_{0}}}$ | $\left( Eq.S9 \right)$ |

where $+$ and $-$ correspond to the cation and anion, respectively, and $D$ is the ion diffusivity. $\zeta_{p}$ is the zeta potential of the particle, and $\kappa^{-1}$ is the characteristic length of the electrical double layer (also known as the Debye length, $\lambda_{D}$).

$K_{sl}$, on the other hand, is typically assigned, and we have chosen a value of 5×10^-9^ S, well within the common range used in the literature[5,6].

The values used in calculating Re (K) are listed in Table S1.

Table S1 Parameters for calculating Re(K)

| Parameter | Description | Value |
| --- | --- | --- |
| *T* | Temperature | 293.15 K |
| $k_{B}$ | Boltzmann constant | 1.38 × 10^-23^ J·K^-1^ |
| *e* | Charge on a proton | 1.6 × 10^-19^ |
| $\varepsilon_{m}$ | Electrical permittivity of water | 78.5ε_0_ |
| $\varepsilon_{{SiO}_{2}}$ | Electrical permittivity of SiO_2_ | 3.9ε_0_ |
| $\varepsilon_{Ag}$ | Electrical permittivity of Ag | 1×10^9^ F·m^-1^ |
| $\varepsilon_{0}$ | Electrical permittivity of vacuum | 8.85 × 10^-12^ F·m^-1^ |
| $\sigma_{{SiO}_{2}}$ | Bulk conductivity of SiO_2_ | 1×10^-10^ S·m^-1^ |
| $\sigma_{Ag}$ | Bulk conductivity of Ag | 6.3×10^6^ S·m^-1^ |
| $\zeta_{p}$ | Particle zeta potential | -50 mV* |
| $D^{+}$ | Diffusivity of H^+^ | 6.22 × 10^-9^ m^2^·s^-1^ |
| $D^{-}$ | Diffusivity of OOH^-^ | 0.3 × 10^-9^ m^2^·s^-1^ |
| $\eta$ | Dynamic viscosity of water at 293.15 K | 1.0016 × 10^-3^ Pa·s^-1^ |
| $K_{sl}$ | Stern layer conductivity | 5×10^-9^ S |

* A zeta potential value of -50 mV was used as a rough value representing the typical zeta potential values of actual microspheres in the range of ~ -36 to -66 mV, shown in Table S1. The choice of this value in the range of -36 mV to -66 mV has a negligible effect on the result of Re(K).

The plots of the real component of the Clausius–Mossotti factor (Re(K)), which represent the polarized direction of particles, are shown as below.


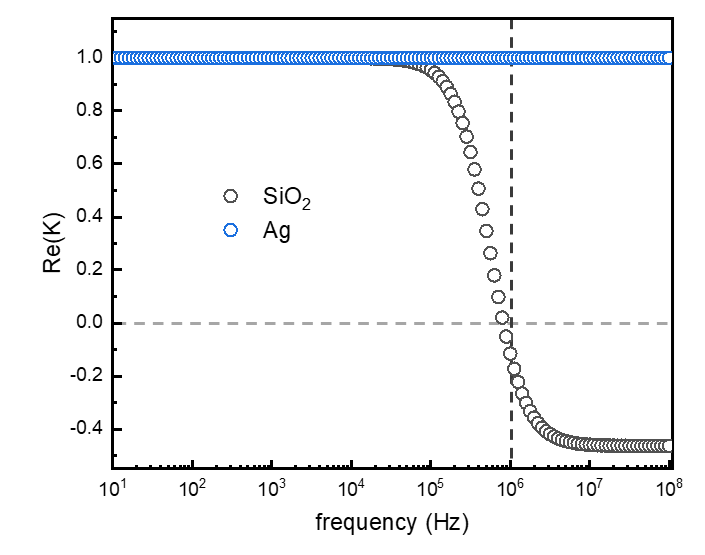


Fig. S4. Plots of the real component of the Clausius–Mossotti factor (Re(K)) as a function of frequency for 3 μm diameter SiO_2_ and 1.5 μm diameter Ag microspheres.

# 3. The whole field of view of colloidal molecules formed


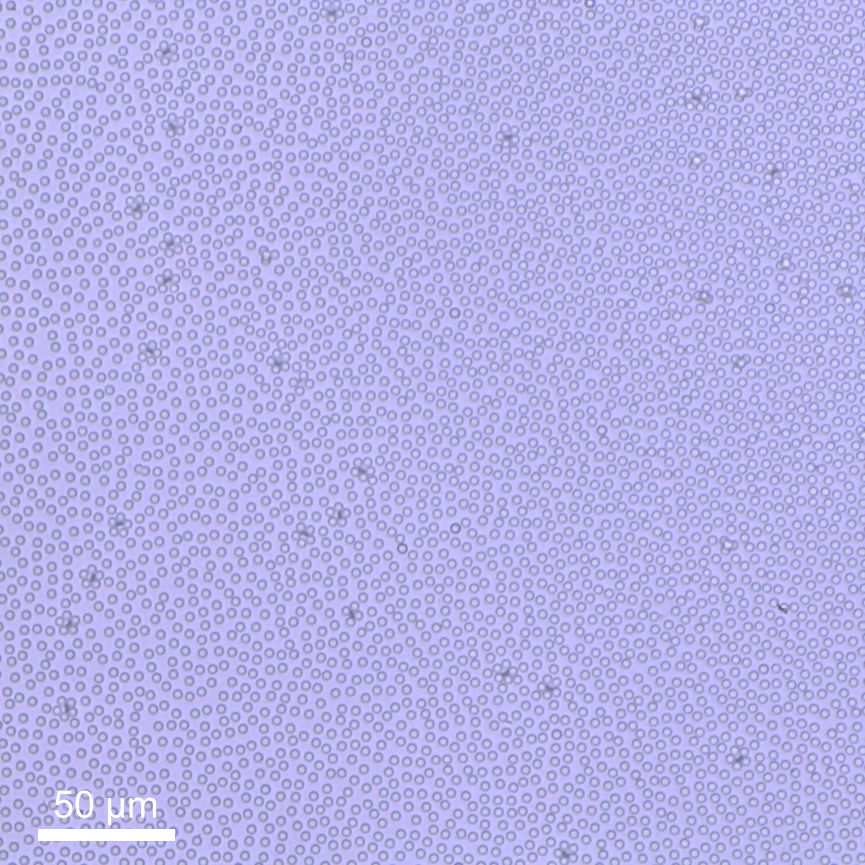


Fig. S5. the whole field of view of colloidal molecules, where 2 μm TiO_2_-SiO_2_ is surrounded by 3 μm SiO_2_ in 1.5 wt% H_2_O_2_ under 563 mW/cm^2^ UV light and AC electric field with 10 V_pp_ and 100 kHz

# 4. Distributions of colloidal molecules with different coordination numbers


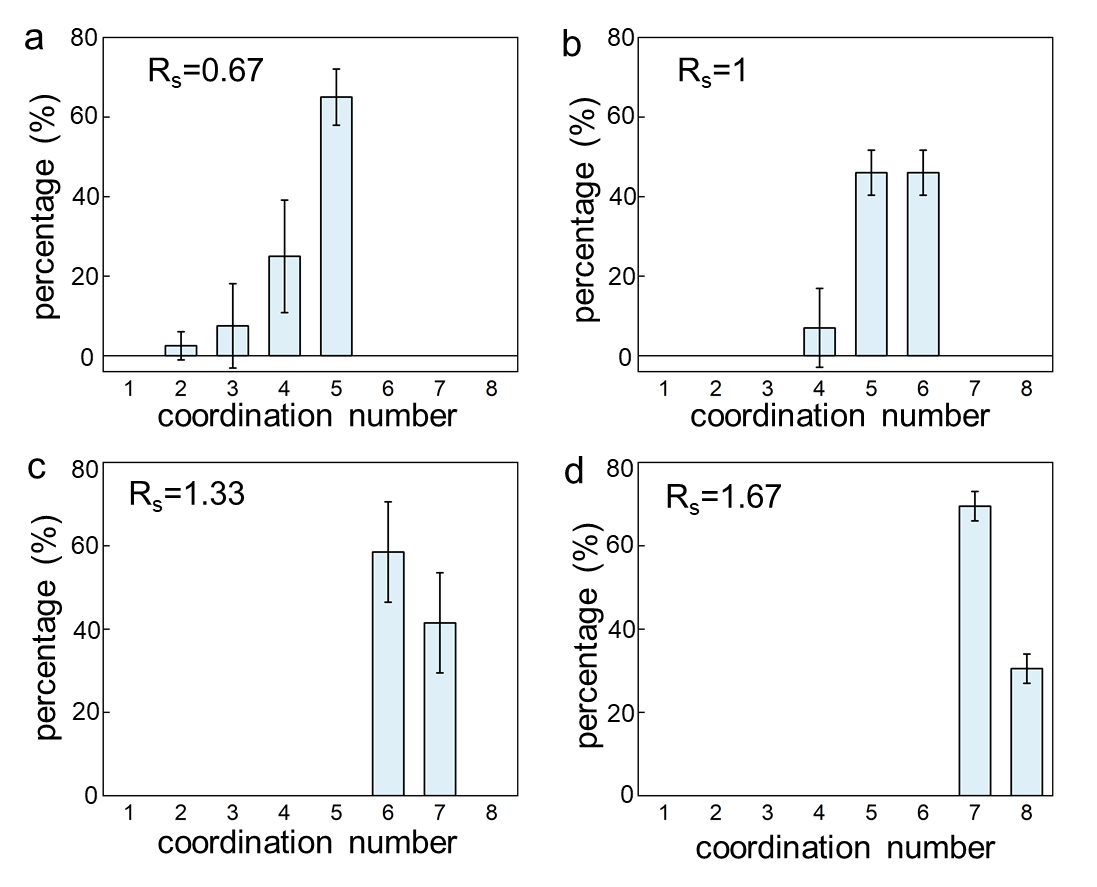


Fig. S6. Distributions of colloidal molecules with different coordination numbers at a) R_s_=0.67, b) R_s_=1, c) R_s_=1.33, d) R_s_=1.67

# 5. Colloidal molecules with different packing fractions


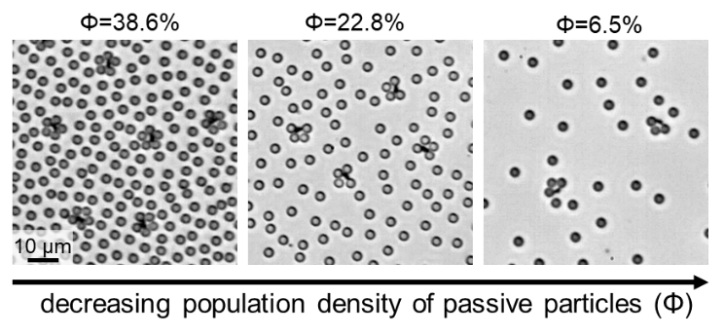


Fig. S7. The formation of colloidal molecules from systems with different population densities of passive particles (packing fraction Φ), where 2 μm TiO_2_-SiO_2_ particles are surrounded by 3 μm SiO_2_ particles in 1.5 wt% H_2_O_2_ under 563 mW/cm^2^ UV and 10 V_pp_ 100kHz AC electric field.

# 6. Transition process of four colloidal molecules


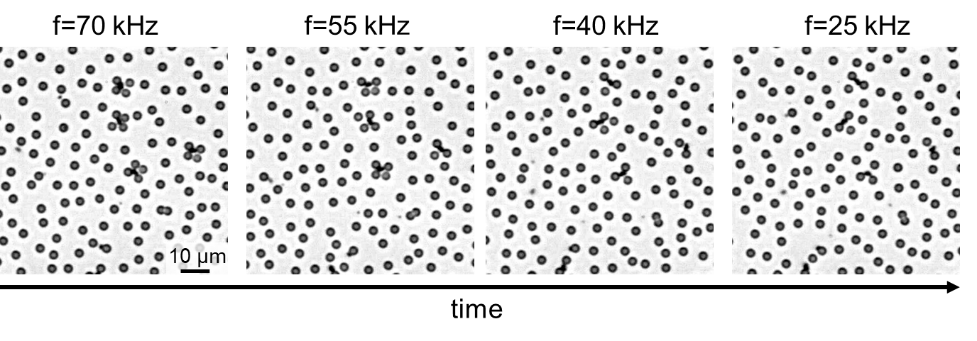


Fig. S8. Transition process of four colloidal molecules by tuning the frequency of the AC electric fields. Experimental conditions: 2 μm TiO_2_-SiO_2_ and 3 μm SiO_2_ under 10 V_PP_ electric field and 563 mW/cm^2^ UV in the 1.5 wt% H_2_O_2_ solution.

# 7. Regulating the structure of colloidal molecules

Under exposure to UV light with an intensity of 563 mW/cm^2^ (100%), the AB_5_ colloidal molecule structure can be observed in the mixture of TiO_2_-SiO_2_ and SiO_2_ particles. When light intensity decreases to 50%, the number of ligands decreases to four. It is worth noting that three ligands are still assembled by the central particles even in the absence of UV light. This can be attributed to the dielectrophoretic effect resulting from the distinct conductivity and electrical permittivity shared by SiO_2_ and TiO_2_ under AC electric field.


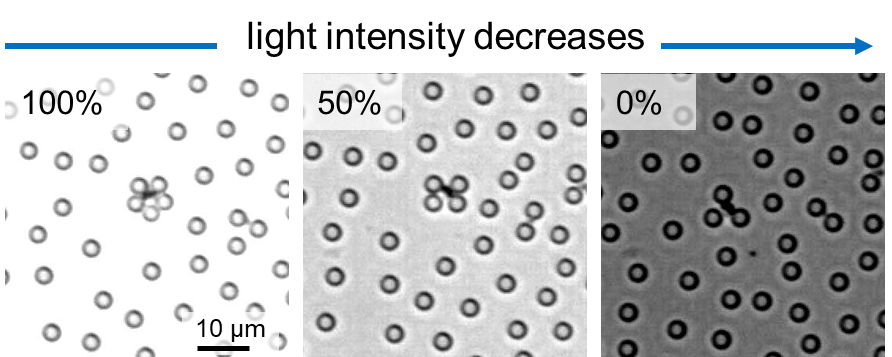


Fig. S9. Regulating the structure of colloidal molecules via changing light intensity. 100% represents for 563 mW/cm^2^.

# 8. Assembled behaviors under only electric field and light, respectively


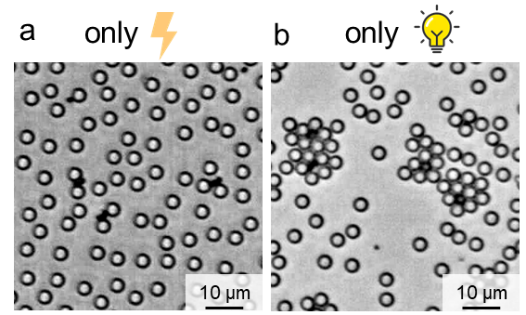


Fig. S10. Assembled behaviors of TiO_2_-SiO_2_ and SiO_2_ particle a) under AC electric field of 1 MHz and 10 V_pp_, b) under 563 mW/cm^2^ UV illumination in 1.5 wt% H_2_O_2_ solution.

# 9. Self-propulsion of a colloidal molecule


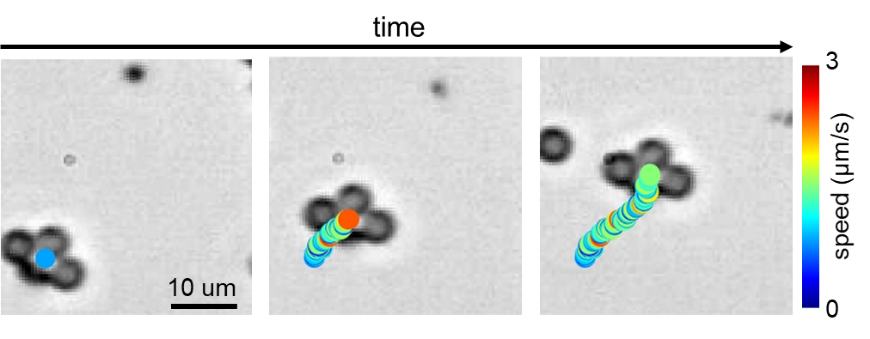


Fig. S11. Trajectory of a colloidal molecule assembled by an active TiO_2_-SiO_2_ and three passive SiO_2_. Its instantaneous speeds are color-coded. Experimental conditions: 2 μm TiO_2_-SiO_2_ and 3 μm SiO_2_ under 10 V_PP_ electric field and 563 mW/cm^2^ UV in the 1.5 wt% H_2_O_2_ solution.

# 10. Self-assembly structures in three dimensions


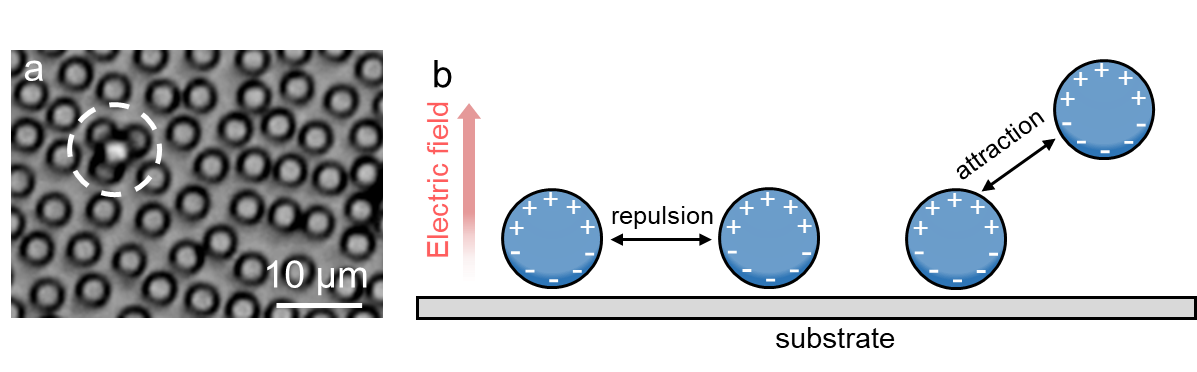


Fig. S12. a) A self-assembly structure in three dimensions; b) the schematic of in-plane repulsion and out-of-plane attraction induced by dipolar interaction under electric field.

# 11. Colloidal assembly with and without long-range term of chemical attraction


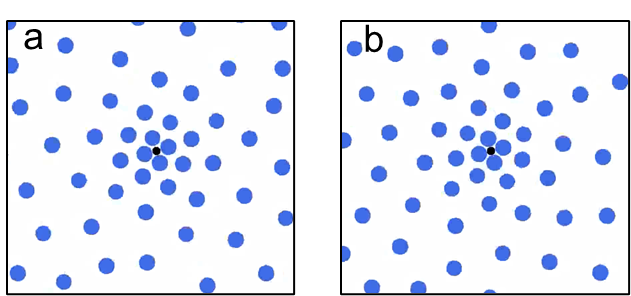


Fig. S13. Colloidal assembly in the BD simulation. a) with long-range term and b) without long-range term of chemical interaction.

# 12.Supporting videos

Video S1. Assembling process of colloidal molecules with Ag and SiO_2_ microspheres as central and ligand particles, respectively. Experimental conditions: 0.01 wt% H_2_O_2_ and AC electric field of 1 MHz and 10 V_pp_.

Video S2. Experimental observation of Ag and SiO_2_ particles without chemical attraction and with dipolar attraction. Experimental conditions: DI water and AC electric field of 1 MHz and 10 V_pp_.

Video S3. Assembling process of colloidal molecules with TiO_2_-SiO_2_ and SiO_2_ microspheres as central and ligand particles, respectively. Experimental conditions: 1.5 wt% H_2_O_2_, 563 mW/cm^2^ UV light and AC electric field of 1 MHz and 10V_pp_.

Video S4. Regulating structural shape of colloidal molecules with TiO_2_-SiO_2_ and SiO_2_ microspheres as central and ligand particles, respectively, by tuning the size ratio of central and ligand particles. Experimental conditions: 1.5 wt% H_2_O_2_, 563 mW/cm^2^ UV light and AC electric field of 1 MHz and 10 V_pp_.

Video S5. Regulating coordination number of colloidal molecules with TiO_2_-SiO_2_ and SiO_2_ microspheres as central and ligand particles, respectively, in real time by tuning the frequency of the electric field from 100 kHz to 25 kHz. Experimental conditions: 1.5 wt% H_2_O_2_, 563 mW/cm^2^ UV light and AC electric field of 10 V_pp_.

# 13. Reference

[1] R. Pethig, Dielectrophoresis: Status of the theory, technology, and applications, Biomicrofluidics 4 (2010).

[2] L.I. L. Alvarez, M. A. Fernandez-Rodriguez, A. Alegria, S. Arrese-Igor, K. Zhao, M. Kröger, Reconfigurable artificial microswimmers with internal feedback, Nat. Commun. 12 (2021) 4762.

[3] V.N. Shilov, A. V Delgado, F. Gonzalez-caballero, C. Grosse, Thin double layer theory of the wide-frequency range dielectric dispersion of suspensions of non-conducting spherical particles including surface conductivity of the stagnant layer, Colloids Surfaces A Physicochem. Eng. Asp. 192 (2001) 253–265.

[4] X. Yang, S. Johnson, N. Wu, The Impact of Stern-Layer Conductivity on the Electrohydrodynamic Flow Around Colloidal Motors under an Alternating Current Electric Field, Adv. Intell. Syst. 1 (2019) 1900096.

[5] R. Pethig, Where is dielectrophoresis (DEP) going?, J. Electrochem. Soc. 164 (2016) B3049.

[6] F. Ma, X. Yang, H. Zhao, N. Wu, Inducing propulsion of colloidal dimers by breaking the symmetry in electrohydrodynamic flow, Phys. Rev. Lett. 115 (2015) 208302.

[7] Y. Mu, L. Lei, J. Zheng, W. Duan, Z. Wang, J. Tang, Y. Gao, Y. Wang, Binary Phases and Crystals Assembled from Active and Passive Colloids, ACS Nano 16 (2022) 6801–6812.

[8] J. Gong, N. Wu, Electric-Field Assisted Assembly of Colloidal Particles into Ordered Nonclose-Packed Arrays, Langmuir 33 (2017) 5769–5776.

[9] X. Yang, N. Wu, Change the Collective Behaviors of Colloidal Motors by Tuning Electrohydrodynamic Flow at the Subparticle Level, Langmuir 34 (2018) 952–960.

[10] S. Zhang, N. Shakiba, Y. Chen, Y. Zhang, P. Tian, J. Singh, M.D. Chamberlain, M. Satkauskas, A.G. Flood, N.P. Kherani, S. Yu, P.W. Zandstra, A.R. Wheeler, Patterned optoelectronic tweezers: A new scheme for selecting, moving, and storing dielectric particles and cells, Small 14 (2018) 1803342.
